# Southeastern Asia fall armyworms are closely related to populations in Africa and India, consistent with common origin and recent migration

**DOI:** 10.1038/s41598-020-58249-3

**Published:** 2020-01-29

**Authors:** Rodney N. Nagoshi, Ni Ni Htain, Duncan Boughton, Lei Zhang, Yutao Xiao, Benjamin Y. Nagoshi, David Mota-Sanchez

**Affiliations:** 1 Center for Medical, Agricultural and Veterinary Entomology, United States Department of Agriculture-Agricultural Research Service, Gainesville, Florida United States of America; 2 Biological Control Section, Plant Protection Division, Yangon, Myanmar; 3 Department of Agricultural, Food and Resource Economics, Michigan State University, East Lansing, Michigan United States of America; 4 Agricultural Genomics Institute at Shenzhen, Chinese Academy of Agricultural Sciences, Shenzhen, 518120 China; 5 University of South Florida, Tampa, Florida United States of America; 6 Department of Entomology, Michigan State University, East Lansing, Michigan United States of America

**Keywords:** Animal migration, Invasive species, Haplotypes, Agricultural genetics

## Abstract

The discovery of fall armyworm, a native of the Western Hemisphere, in western Africa in 2016 was rapidly followed by detections throughout sub-Saharan Africa, India, and most recently southeastern Asia. This moth pest has a broad host range that threatens such important crops as corn, rice, millet, and sorghum, creating concern for its potential impact on agriculture in the Eastern Hemisphere. Although genetic data suggest populations sampled in Africa and India originate from a recent common source, it is not known whether this is the case for populations in southeastern Asia, nor whether the subgroup with a preference for rice and millet is present in the region. This study found through comparisons of genetic markers that the fall armyworm from Myanmar and southern China are closely related to those from Africa and India, suggesting a common origin for these geographically distant populations. The results are consistent with a single recent introduction into the Eastern Hemisphere followed by rapid dispersion. The molecular similarities include discrepancies between the genetic markers that brings into question whether the subpopulation most likely to be a threat to rice and millet is present in significant numbers in Asia.

## Introduction

The fall armyworm (*Spodoptera frugiperda*; J. E. Smith)(Lepidoptera: Noctuidae) is a significant economic pest of corn and other crops in the Western Hemisphere and is noted for its broad host range (over 80 host plant species reported) and long-distance migration capability^1^. These characteristics have become a global concern with the introduction of fall armyworm into the Eastern Hemisphere and its spread from western Africa to southeastern Asia over a remarkably short period of time. Large infestations of fall armyworm in Africa were first reported in southwestern Nigeria in 2016^2^. Within the next two years infestations were observed in most sub-Saharan nations ranging from Kenya to the east and South Africa to the south^3–7^. In 2018 fall armyworm populations were found in multiple locations in India^8–11^, and now most recently in southeastern Asia^12–14^.

Conjectures about fall armyworm movements in the Eastern Hemisphere depend on whether the timing of first detections in various regions accurately reflect the first arrival of the pest. If so, then fall armyworm in two years traversed a minimum of 7,000 km from Nigeria to India and then another 4,000 km in the next year to southeastern Asia. If this occurred through natural migration it would seemingly require movements over large bodies of water, desert, and other habitats where the primary host plants would be expected to be scarce. Fall armyworm does migrate thousands of kilometers annually in North America, but this occurs with very favorable wind patterns and a plentiful supply of corn acreage along the migration routes^15^. It therefore seems likely that if the dispersion of fall armyworm in the Eastern Hemisphere began in 2016 from a western Africa entry point, human transport and commerce played a significant role to facilitate the movements. If correct, this scenario would indicate a remarkable susceptibility of the Eastern Hemisphere to invasions by exotic migratory moth pests. Understanding how such population movements occurred will become critical to mitigating future such occurrences.

An alternative possibility is that fall armyworm has long been endemic in the Eastern Hemisphere but was undetected until 2016. In this case, the enhanced monitoring occurring as a consequence of its discovery is giving the illusion of rapid migration. However, the limited amount of genetic variation found in the Eastern Hemisphere populations so far tested and the genetic homogeneity between the fall armyworm in India and Africa are not compatible with this proposal^16^. Instead, they suggest a recent and common origin for these geographically distant populations. Whether this is also the case with the southeastern Asia fall armyworm populations has not been tested to our knowledge.

A second area of concern and uncertainty is whether both strains of fall armyworm are present in the Eastern Hemisphere and in particular Asia. The broad host range exhibited by the species is in part due to the presence of two subpopulations that differ in their host plant preferences. Originally labelled as “host strains” the groups were named after the crop upon which they were first identified, with the “rice-strain” in subsequent studies preferentially found in pasture grass and millet, while the “corn-strain” predominates in corn and sorghum^17–19^. Although initially identified on rice, the specificity of the rice-strain to this host appears to be more variable and therefore uncertain^20^. Because the host specificities of the two strains are still being determined we will from this point refer to the corn-strain and rice-strain by the more generic terms C-strain and R-strain, respectively.

A complicating factor particularly in field studies is that the strains are for all practical purposes morphologically indistinguishable, with molecular markers the most reliable diagnostic tool. Specifically, the biased host plant distribution of the C-strain and R-strain populations show a consistent but not absolute correspondence with genetic polymorphisms in the mitochondrial *cytochrome oxidase subunit I* (*COI)* and nuclear *triosephosphate isomerase* (*Tpi)* genes that themselves are generally, but not always, in agreement^17,21,22^. Typically about 20% of larvae collected directly from corn plants and adult males from pheromone traps placed in corn fields display R-strain diagnostic molecular markers^23–25^.

An unusual feature of the collections so far analyzed from Africa and India is that as defined by the *COI* marker the R-strain is the predominant form in most locations even though all collections tested to date came from C-strain preferred hosts^5,6,16^. However, when the same collections were tested for the *Tpi* marker, >95% of the specimens were identified as C-strain. The correspondence of the *Tpi* identification with host plant and its disagreement with *COI* suggests that *COI* may not be an accurate strain marker in Africa and India. If true then the presence of the R-strain is in question in the Eastern Hemisphere, an important consideration for risk assessments given the importance of R-strain preferred crops in many Asian countries.

There are two objectives to this study. The first is to assess the similarity of the fall armyworms found in southeast Asia with those from Africa and India to estimate the likelihood that they are part of the same invasion event. The second is to determine whether the disagreement between the *COI* and *Tpi* strain markers observed in Africa and India is also a characteristic of southeast Asian fall armyworm. We discuss the implications of the results to our understanding of fall armyworm movements in the Eastern Hemisphere and the risk posed by this pest on R-strain preferred crops (such as rice and millet) in Asia.

## Methods

### Specimen collections and dna preparation

**Figure 1:**
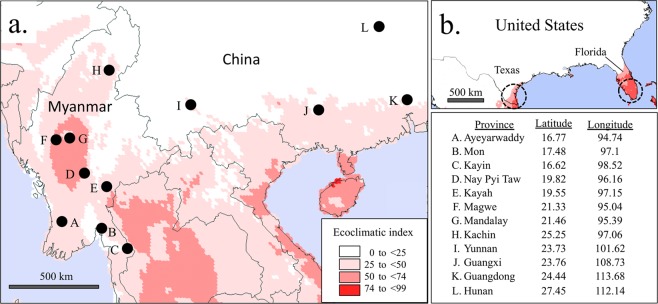
Map and coordinates of collection sites in Myanmar and China combined with CLIMEX modeling of area suitability for fall armyworm. Collection (i) describes pheromone trapping of adult males. All others represent larval collections from corn host plants. (**a**) locations of sites overlaid on CLIMEX projection of fall armyworm suitability based on calculations of the Ecoclimatic Index (EI), with higher values indicating greater likelihood of persistent fall armyworm populations. (**b**) CLIMEX projections for the southeastern United States with the same parameters used in Asia. Circles indicate approximate regions where fall armyworm populations are localized during the winter in the United States based on monitoring studies.

Larval collections were made in 2018 from eight provinces in Myanmar and subdivided into three groups approximating lower Myanmar (Ayeyarwaddy, Mon, and Kayin), upper Myanmar (Nay Pyi Taw, Kayah, Magwe, and Mandalay), and hilly regions (Kachin). Identification of fall armyworm specimens was performed using morphological criteria^14^. In a separate survey of China, collections were made by pheromone trapping of adult males in Yunnan province and larval collections in Guangxi, Guangdong and Hunan provinces during March to May in 2019 (Fig. 1a). Collected specimens were stored dry or in ethanol. There are numerous lepidopteran pests of corn reported in southeastern Asia that potentially complicates the identification of fall armyworm^26^. Therefore, fall armyworm identity for all Asian specimens was confirmed by *COI* sequence analysis. Collections and data from previous studies include larval collections from Florida^21^, Argentina^17^, India^16^, and Africa^5^.

Larvae from Myanmar were processed using a 5-ml Dounce homogenizer (Thermo Fisher Scientific, Waltham, MA, USA) in 800 µl Genomic Lysis buffer (Zymo Research, Orange, CA, USA). The homogenate was incubated at 55 °C for 15–30 min, then centrifuged at 10,000 rpm for 5 min. DNA was purified using a Zymo-Spin III column (Zymo Research, Orange, CA, USA) and processed according to manufacturer’s instructions. Genomic DNA preparations were stored at −20 °C. Species identity was initially estimated by larval morphology and confirmed by *COI* sequence analysis.

Larvae from China were ground with liquid nitrogen and genomic DNAs extracted with Axyprep Multisource Genomic DNA Miniprep Kit (Corning, Corning, NY) according to manufacturer’s instructions. Genomic DNA were stored at −20 °C before analysis. Species were identified according to morphological characteristics.

### PCR amplification and DNA sequencing

Polymerase chain reaction (PCR) amplification occurred in a 30 µl reaction mix with a final concentration of 1X ThermoPol reaction buffer and 0.75 units Taq DNA polymerase (both from New England Biolabs, Beverly, MA), 0.17 mM dNTP, and 0.3 µM of each primer. The PCR protocol was 94 °C (1 min), followed by 30 cycles of 92 °C (30 s), 58 °C (30 s), 72 °C (45 s), and a final segment of 72 °C for 3 min. Primers were synthesized by Integrated DNA Technologies (Coralville, IA). Amplification of the COIB segment was done with primers *c924F* (5′-TTATTGCTGTACCAACAGGT-3′) and *c1303R* (5′- CAGGATAGTCAGAATATCGACG-3′). Samples that gave poor amplification were reanalyzed using nested PCR in which the first amplification was performed using primers *c891F* (5′-TACACGAGCATATTTTACATC-3′) and *c1472R* (5′-GCTGGTGGTAAATTTTGATATC-3′) followed by a second PCR using the internal primers *c924F* and *c1303R*. Amplification of the *Tpi* segment was done with primers *t412F* (5′- CCGGACTGAAGGTTATCGCTTG -3′) and *t1140R* (5′- GCGGAAGCATTCGCTGACAACC-3′). Nested PCR was also used, with the first PCR done with primers *t634F* (5′-TTGCCCATGCTCTTGAGTCC-3′) and *t1166R* (5′-TGGATACGGACAGCGTTAGC-3′) and the second PCR using the internal primers *t412F* and *t1140R*.

For gel electrophoresis, 6 µl of 6X gel loading buffer was added to each amplification reaction and the entire sample run on a 1.8% agarose horizontal gel containing GelGreen (Biotium, Hayward, CA) in 0.5X Tris-borate buffer (TBE, 45 mM Tris base, 45 mM boric acid, 1 mM EDTA pH 8.0). Fragments were visualized on a blue light box and excised from the gel. DNA purification was performed using Zymo-Spin I columns (Zymo Research, Orange, CA) according to manufacturer’s instructions. Genewiz (South Plainfield, NJ) performed the DNA sequencing.

DNA alignments and consensus building were performed using MUSCLE (multiple sequence comparison by log-expectation), a public domain multiple alignment software. Phylogenetic trees were constructed using the Tamura-Nei genetic distance model and the UPGMA tree building network^27^. These programs are incorporated into the Geneious Pro 10.1.2 program (Biomatters, New Zealand, http://www.geneious.com)^28^.

### Characterization of the *COI* and *Tpi* gene segments

The *COI* and *Tpi* strain diagnostic markers are single nucleotide substitutions. Site designations begin with an “m” (mitochondria) or “g” (genomic). This is then followed in order by the gene name, number of base pairs from the predicted translational start site (for *COI*) or the 5′ start of the exon (*Tpi*), and finally the observed polymorphism using IUPAC convention (R = A or G; Y = C or T; W = A or T; K = G or T; S = C or G; D = A or G or T).

**Figure 2:**
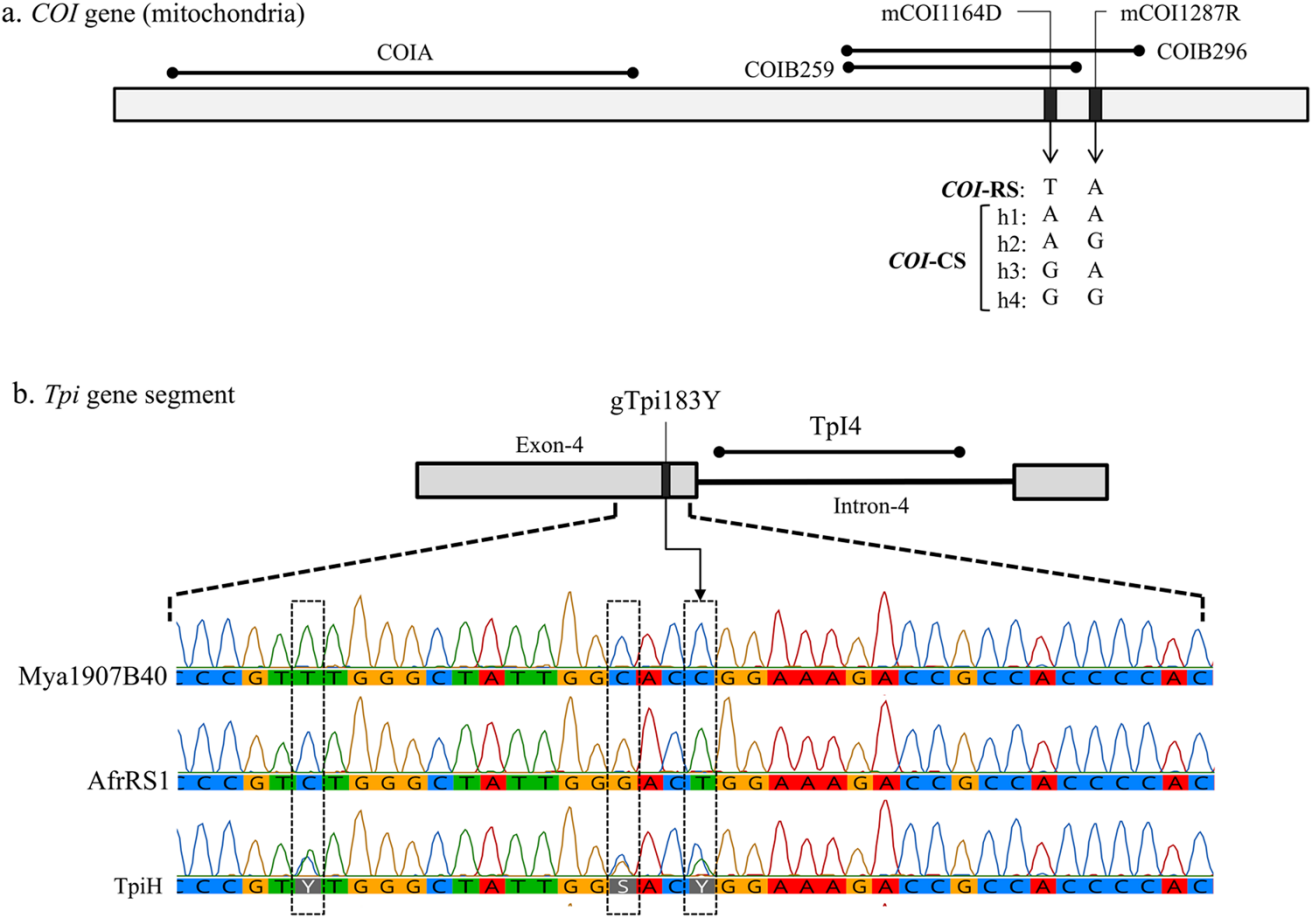
Diagrams of relevant regions in the *COI* and *Tpi* genes used for molecular analysis. (**a**) the *COI* gene segments with locations of polymorphic sites used for categorizing the *COI*-CS h1-h4 variants. (**b**) Map of the *Tpi* gene segment consisting of the fourth exon of the presumptive open reading frame and adjacent intron. Site gTpi183Y defines the Tpi-based strain identity. Below are chromatographs for the exon segment containing gTpi183Y and two other strain-specific polymorphic sites. Mya1907B40 is a TpiC allele found in Myanmar while AfrRS1 is the TpiR allele identified in Africa. Combining the two produces the overlapping chromatograph pattern found with TpiH.

The COIB segment was amplified by primers *c924F* and *c1303R*. Species identity of the Myanmar specimens was confirmed by sequence comparisons of COIB259, a 259-bp segment common to the GenBank sequences for the following *Spodoptera* species, *S. abula* (HQ177287), *S. cosmiodes* (HQ177295), *S. descoinsi* (HQ177306), *S. dolichos* (HQ177313), *S. eridania* (Stoll in Cramer)(HQ177321), *S. exempta* (Walker)(HQ177334), *S. exigua* (Hübner)(HQ177339), *S. latisfscia* (Walker)(HQ177354), *S. littoralis* (Boisduval)(HQ177364), *S. mauritia* (Boisduval)(HQ177382), *S. ornithogalli* (Guenée)(HQ177392), *S. praefica* (Grote)(HQ177407), *S. litura* (F.)(HQ177375). Sites mCOI1164D and mCOI1287R are diagnostic for strain identity in Western Hemisphere populations where there is a single rice-strain, T_1164_A_1287_, and four corn-strain configurations, A_1164_A_1287_ (h1), A_1164_G_1287_ (h2), G_1164_A_1287_ (h3), and G_1164_G_1287_ (h4)^29^ (Fig. 2a).

The *Tpi* Exon-4 segment consists of multiple strain specific polymorphisms with the gTpi183Y site considered diagnostic of strain identity (Fig. 2b). A C_183_ identifies the C-strain allele, TpiC, while T_183_ defines the R-strain, TpiR^21^. The *Tpi* gene is located on the *Z* sex chromosome that is present in one copy in females and two copies in males, with the latter providing opportunities for heterozygosity. Because the genomic DNA was directly sequenced, males heterozygous for *Tpi* alleles will simultaneously display both alternatives at polymorphic sites, which if different can be identified by overlapping sequencing chromatographs. Heterozygosity at site gTpi183Y gave rise to an overlapping C and T signal at gTpi183Y. This was designated TpiH and defined as representing a TpiC/TpiR heterozygote.

A portion of the adjacent *Tpi* intron was previously used in phylogenetic comparisons^30,31^. An approximately 172-bp segment of the intron (TpI4) beginning 10-bp from the 5′ splice site was used in the analysis, with the length variable because of indels. This segment was chosen because it displayed the most consistent sequence quality with the given primers. Many samples examined were heterozygous for frameshift mutations within the intron that produced overlapping chromatographs beginning at the polymorphism. These were not further analyzed. Sequences deposited in GenBank include Mya1907c28 (MN551267), Mya1910a85 (MN551268), Mya1907d14 (MN551269), Mya1910B28 (MN551270), AfrCa1a (MN551271), AfrCa2a (MN551272), AfrCa2b (MN551273), AfrCa1b (MN551274), AfrCa2c (MN551275), and AfrRa1a (MN551276).

### Calculation of haplotype numbers

Specimens have a single mitochondrial *COI* haplotype and so frequency was calculated as the number of specimens with a given *COI* haplotype divided by the total number of specimens. The *Tpi* marker is more complicated because of the potential for heterozygosity. Specimens can be characterized by three *Tpi* strain categories, TpiC (C-strain), TpiR (R-strain), and TpiH (TpiC/TpiR heterozygote). Frequencies at the specimen level were calculated by the number of each category divided by the total number of specimens. We also calculated the frequency of *Tpi* chromosomes, which allows inclusion of the TpiH specimens when estimating the number of *Tpi* alleles. Larvae were not sexed so those identified as TpiC or TpiR could have one (females) or two (males) copies of the *Tpi* gene. We accounted for this uncertainty by assuming a 1:1 sex ratio and using 1.5 as the mean number of Tpi genes per TpiC or TpiR specimen based on the formula of [2 (*Tpi* genes in males) + 1 (*Tpi* gene in females)]/2. The TpiH specimens were presumed to carry one copy each of TpiC and TpiR. From these considerations we derived the following formulae, TpiC (chromosomes) = 1.5 X TpiC (specimens) + TpiH and TpiR (chromosomes) = 1.5 X TpiR (specimens) + TpiH. Chromosome frequency was calculated by dividing the number of TpiC or TpiR chromosomes by the total number of chromosomes, as determined by the equation Total chromosomes = 1.5(TpiC + TpiR specimens) + 2(TpiH specimens).

### CLIMEX climate suitability analysis

CLIMEX estimates the potential geographical distribution and relative abundance of a species based on biological parameters and regional climate conditions^32^. The biological parameter values for fall armyworm were previously published (Table 1)^33^. Climate information was imported from Climond (www.climond.org)^32,34^ for selected regions using historical data from 1961–1990 at a resolution of 10 feet.

**Table 1:** CLIMEX parameter values used for modelling fall armyworm.

Parameter	Description	Value
Moisture		
SM0	Lower soil moisture threshold	0.1
SM1	Lower optimal soil moisture	0.7
SM2	Upper optimal soil moisture	0.9
SM3	Upper soil moisture threshold	1.5
0.5Temperature		
DV0	Lower temperature threshold	12 °C
DV1	Lower optimal temperature	22 °C
DV2	Upper optimal temperature	27 °C
DV3	Upper temperature threshold	34 °C
Cold Stress		
TTCS	Cold stress temperature threshold	8 °C
THCS	Cold stress accumulation rate	−0.001 week^−1^
Heat Stress		
TTHS	Heat stress temperature threshold	38 °C
THHS	Heat stress accumulation rate	0.001 week^−1^
Dry Stress		
SMDS	Soil moisture dry stress threshold	0.1
HDS	Dry stress accumulation rate	−0.001 week^−1^
Wet Stress		
SMWS	Soil moisture wet stress threshold	1.5
HWS	Wet stress accumulation rate	0.001 week^−1^
Minimum degree-day sum needed to complete a generation		
PDD	Degree-Days per generation	559 °C

The Ecoclimatic Index (EI) integrates projected growth potential counterbalanced by estimates of stress, the latter of which is based primarily on unfavorable temperature and moisture conditions. EI is presented on a 0–100 scale, where 100 represent continuous 100% suitability (as in an incubator). For this study, the Compare Locations (1 species) function in the CLIMEX program was used with the Grid Data simulation file. No climate change scenario or irrigation components were set. An EI map was created from the simulation.

## Results

### Climate suitability projections

Locations with climatic conditions suitable for fall armyworm populations in the surveyed region were determined by CLIMEX analysis (Fig. 1a). Ecoclimatic index (EI) values were calculated with areas with an EI equal to or greater than 25 indicated in red with darker shading indicating higher values. The higher the EI value, the greater the likelihood of persistent fall armyworm populations, with values greater than 30 considered to be high suitability^32^. All but one collection site was in the vicinity of suitable habitats. To assess the accuracy of the CLIMEX analysis the same parameters were used to analyze the southeastern United States for fall armyworm suitability. Two United States locations were identified, in southern Texas and Florida (Fig. 1b), approximately consistent with estimates of the overwintering range derived from pest monitoring^1,35^. These results indicate that the surveyed locations include areas with a high probability of supporting permanent fall armyworm populations and so are potential sources of migratory populations.

### Characterization of Myanmar fall armyworm using *COI*

**Figure 3:**
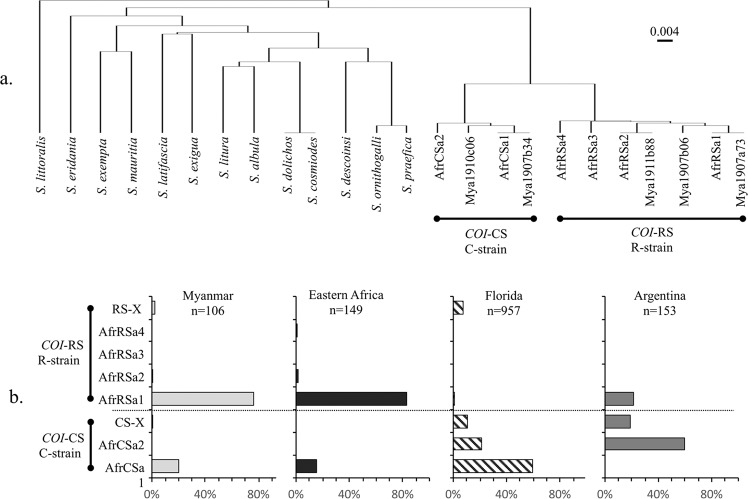
Phylogenetic tree calculated for the COIB259 sequence using the UPGMA method and Tamura-Nei modeling together with comparisons of haplotype frequencies from different regions. (**a**) Comparison of COIB259 haplotypes from Myanmar (MyaXXXXXXX) compared to 13 *Spodoptera* species (from GenBank) and haplotypes observed in Africa (AfrXXXX). (**b**) *COI*-CS variants that differ from those observed in Africa.

The mitochondrial *COI* and nuclear *Tpi* genes carry polymorphisms used to characterize fall armyworm populations (Fig. 2). Sequence analysis of the COIB259 segment (COIB259) from *COI* identified five haplotypes from 106 specimens collected from eight provinces in Myanmar. Phylogenetic comparisons with sequences from 13 Spodoptera species confirmed the fall armyworm identification of the Myanmar specimens and identified two C-strain (*COI*-CS) and three R-strain (*COI*-RS) variants (Fig. 3a). Three Myanmar COIB sequences (Mya1907a73, Mya1911b88, and Mya1907b34) are identical to haplotypes found in Africa, while Mya1907b06 and Mya1910c06 differ by only a single base change from the closest Africa variants.

The two most common Myanmar COIB259 haplotypes, Mya1907a73 (AfrRSa1) and Mya1907b34 (AfrCSa1) accounted for more than 96% (102/106) of the Myanmar collections (Fig. 3b). These are also the most frequent haplotypes in Africa, where the relative proportions observed in pooled collections from eastern Africa (Burundi, Kenya, and Tanzania) were similar to that observed in Myanmar.

Although the Myanmar collections were from corn only a minority of 19% (22/106) displayed the *COI*-CS haplotype of the C-strain that is associated with corn preference. This differs from what is typically observed in the Western Hemisphere as indicated by pooled collections from corn hosts in Florida and Argentina (Fig. 3b). A comparison of these collections reveals a regional haplotype bias where AfrCSa1 is the majority *COI*-CS haplotype in Florida while AfrCSa2 predominates in Argentina. All 22 of the *COI*-CS specimens so far examined from Myanmar are AfrCSa1. Additional sequence analysis was performed (COIB296) to allow analysis of sites mCOI1164D and mCOI1287R that in combination produce sequence variants with geographical differences in distribution (h1-h4, Fig. 2a)^36^. All *COI*-CS specimens from Myanmar expressed the h4 combination of G_1164_G_1287_, which is the subgroup most commonly found in Florida and the Caribbean^29^ and a result similar to that observed in India and Africa^16,30^.

### Characterization of Myanmar fall armyworm using *Tpi*

**Figure 4:**
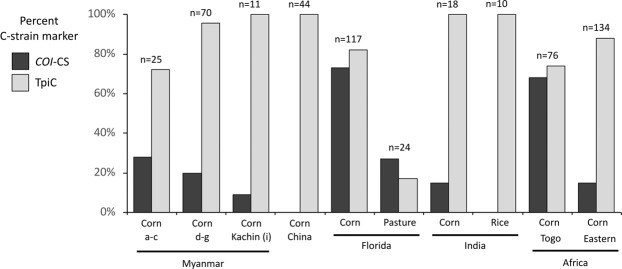
Comparisons of C-strain marker frequencies of larval collections from different locations and host plants. Myanmar sites were grouped by region with letter designations defined in Fig. 1. Eastern Africa is represented by pooled data from Burundi, Kenya, and Tanzania.

The distributions of the *COI* and *Tpi* strain markers in the Western Hemisphere are exemplified by larval collections from Florida^21^ where the C-strain *COI*-CS and TpiC markers generally predominate in specimens from corn and sorghum but are a minority in R-strain hosts such as pasture grasses (Fig. 4). In the Myanmar collections, pooled data from the southern provinces (A-C from Fig. 1a), central provinces (D-G), and Kachin province (I) showed a majority *COI*-RS and TpiC expression, a pattern also found in eastern Africa and India (Fig. 4).

**Figure 5:**
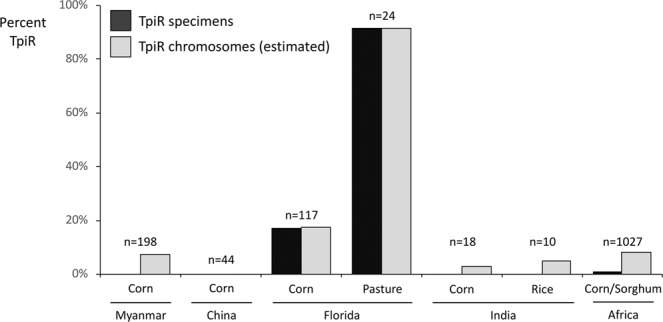
Frequencies of the TpiR haplotype from different regions and host plants calculated on the basis of specimens expressing the haplotype or estimation of chromosome numbers that include contributions from the presumed TpiH interstrain hybrid.

The TpiR sequence was not directly detected in the Myanmar collections (Fig. 5). This included an additional 92 specimens analyzed for the *Tpi* markers for a total sample size of 198. The great majority of specimens were TpiC (169) and with the remainder TpiH.^29^ However, it is likely that the TpiH class is made up of heterozygotes carrying both TpiR and TpiC. This is indicated by the TpiH DNA sequence chromatographs where the pattern of overlapping signals that can be explained by the presence of both TpiC and TpiR haplotypes (Fig. 2b). In this specific case, overlaying the most common TpiC haplotype in Myanmar with the most frequent TpiR haplotype found in Africa (AfrRS1) predicts the overlapping chromatograph pattern of the most frequently observed TpiH pattern. Based on the assumption that TpiH specimens carry one TpiR copy we estimate that TpiR represents about 7% of the *Tpi* chromosomes in Myanmar, a frequency similar to that calculated for fall armyworm in India and Africa but 2–3 times lower than that typically observed in Western Hemisphere collections from C-strain hosts (Fig. 5).

### Comparisons between the African and Myanmar fall armyworms

**Figure 6:**
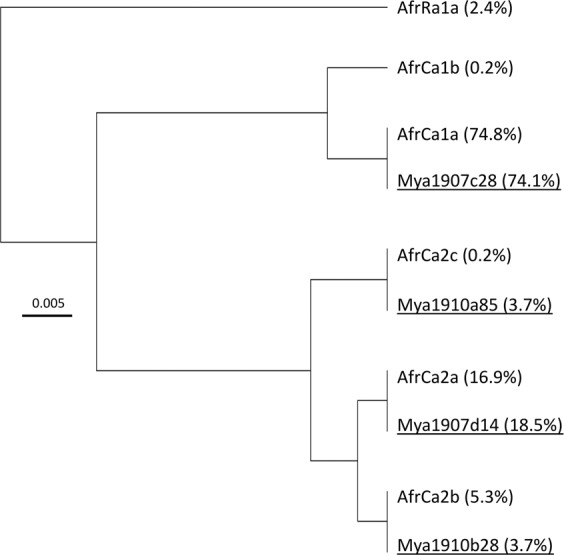
Phylogenetic tree calculated for the TpI4 intron segment using the UPGMA method and Tamura-Nei modeling. Relative frequencies of the different haplotypes are in parentheses.

Adjacent to the strain diagnostic polymorphisms in the fourth exon of the putative *Tpi* coding region is a variable length intron that exhibits high sequence variability in Western Hemisphere populations^31^. The 172-bp segment of this intron identified 138 variants from Western Hemisphere collections compared to only six haplotypes from 863 Africa specimens^30^. From the 207 Myanmar specimens examined for the *Tpi* intron four different haplotypes were identified, each of which is identical to sequences found in Africa (Fig. 6). The frequencies of the haplotypes in the Myanmar collections were similar to that observed in Africa.

### Characterization of fall armyworm from southern China

In a preliminary sampling of southern China, a total of 44 fall armyworm specimens were collected from corn plants in four provinces. In this study the *COI* strain identity was determined using the COIA segment (Fig. 1a)^31^. All specimens were of a single haplotype indicative of *COI*-RS (Fig. 3). Analysis of the segment of the strain diagnostic *Tpi* exon also showed a single sequence corresponding to TpiC (Fig. 4).

## Discussion

The genetic evidence from studies of fall armyworm from multiple locations in Africa, India, and now Myanmar and China indicate that these populations share a common and recent origin that derived from a small number of introductions (as few as one) from the Western Hemisphere. The strongest evidence comes from the examination of a highly variable segment of a *Tpi* intron sequence for which over 200 variants have been identified in the Western Hemisphere. In contrast only four different sequences were found in Myanmar out of 150 specimens tested and these are identical to those found in Africa and India (Fig. 6). Additional supporting evidence comes from the similarities in the types and relative frequencies of the *COI* haplotypes in populations from Myanmar and in particular eastern Africa compared to that typically observed in the Western Hemisphere (Fig. 3b). This includes the predominance of the *COI*-RS marker in collections from C-strain host plants. Finally, the TpiR haplotype found in the TpiH specimens from Myanmar is identical to the single TpiR variant found in Africa, which has so far not been observed in the Western Hemisphere. This combination of genetic similarity between the Eastern Hemisphere fall armyworm populations and low genetic variability is most consistent with a single introduction and subsequent dispersion between Africa and Asia that occurred too recently for the accumulation of sequence polymorphisms. If this scenario is correct it would indicate that the variety of seas, deserts, and mountain ranges that separate Africa and Asia are readily traversed by fall armyworm, suggesting that the hemisphere is at high risk for invasions by migratory moth pests. Estimating the relative contributions of natural and human-assisted (trade and travel) mechanisms for the dispersal of fall armyworm in the Eastern Hemisphere will be essential to assessing the risks of future events of this type and developing effective control strategies.

CLIMEX analysis was used to provide a preliminary estimate of where persistent or permanent fall armyworm populations are most likely to be situated. These could potentially serve as sources for annual migrations in Asia analogous to how North American infestations originate from overwintering locations in south Texas and Florida^15,37^. CLIMEX parameters that produce projections accurately approximating North American overwintering locations show substantial regions in southeastern Asia suitable for fall armyworm populations (Fig. 1a). One collection site in the Hunan province of China (L, Fig. 1a) lies approximately 500 km from suitable habitats as modeled by CLIMEX, suggesting that the fall armyworm found there were likely to be migrants.

Critical to projections of the range of crops at risk is determining to what extent the R-strain is present in Asia as this subpopulation would be the primary fall armyworm threat to such crops as rice and millet. The available genetic markers are contradictory as the *COI*-RS haplotype diagnostic of the R-strain and the TpiC marker indicative of the C-strain predominate in the collections from Africa, India, and from this study, Asia^5,6,16,30^. There are two lines of evidence that suggest that a single strain predominates in the Eastern Hemisphere and that it is most likely the C-strain. The first is that major infestations have been primarily, if not exclusively, reported in the C-strain preferred host plants corn and sorghum, with genetic characterizations to date limited to collections from these sites. Therefore, the TpiC marker is displaying the expected correspondence with host plant preference. Second, a methodology was developed that could detect suppression of interstrain mating in Western Hemisphere field populations by comparing the frequency of heterozygosity between strain-specific genetic polymorphisms with those that are nonspecific^38^. Application of this method to African fall armyworm found no evidence of similar strain-dependent mating behavior^39^. Third, previous studies in the Western Hemisphere indicate that approximately 20% of fall armyworm larvae collected from corn are of the R-strain based on a variety of molecular markers^17,21,25,40^, including TpiR (Fig. 5). In contrast, only 11 TpiR specimens have been detected in the total of 1297 samples so far analyzed from the Eastern Hemisphere. At this time, the only evidence for TpiR in India, Myanmar, and China is from TpiH heterozygotes. Overall, these observations suggest that the African fall armyworm is behaving as expected for the C-strain, with the R-strain a minor presence or perhaps even absent.

In Myanmar, China, India, and most of Africa, the *COI* strain marker is in disagreement with both *Tpi* and host plant. One way this could have occurred is if the linkage between the mitochondrial *COI* marker and strain identity was disrupted by interstrain mating. For example, since mitochondria is maternally inherited, mating between an R-strain female and C-strain male would produce *COI*-RS hybrid daughters, which if they also mated with C-strain males would produce *COI*-RS progeny in a C-strain (including TpiC) background. Under this scenario the originating population entering the Eastern Hemisphere carried both strains with interstrain hybridization occurring as described above. If chance or circumstance caused the R-strain subgroup to diminish then the predominating C-strain would still be associated with TpiC but would now a mixture of the *COI* markers. If correct then the *COI* marker is no longer strain-specific in the Eastern Hemisphere populations, leaving only the TpiC haplotype as a diagnostic molecular marker of strain identity.

Fall armyworm in the Eastern Hemisphere appears to be recently arrived and in the process of rapid dispersion. If so, we can expect substantial changes in haplotype frequency and distribution as populations equilibrate and more extensive and systematic monitoring are performed. The current study provides a snapshot of the surveyed populations in Myanmar and parts of China in 2018–2019, providing a genetic baseline for future comparisons. Of particular interest and economic relevance is whether the R-strain, the fall armyworm subpopulation believed to be the biggest threat to rice and millet, is present in Asia. Even if it is not, the apparent rapidity and extent of the current fall armyworm invasion is a warning of how quickly the R-strain could become widely disseminated in Asia if introduced. Similarly, fall armyworm in the Western Hemisphere can exhibit resistance to several insecticidal proteins from the bacterium *Bacillus thuringiensis* (Bt) that are used in transgenic corn lines^41–43^. At this time, there is no evidence that these resistance traits are present in Africa^6^. These observations underscore the importance of understanding the migratory history of fall armyworm in the Eastern Hemisphere in order to prevent or slow future introductions of the R-strain (if not already present) or other fall armyworm subpopulations from the Western Hemisphere known to carry pesticide resistance or other deleterious traits.

## Data Availability

All data generated or analyzed during this study are included in this published article.
